# MAGIC (maternal glucose in pregnancy) understanding the glycemic profile of pregnancy, intensive CGM glucose profiling and its relationship to fetal growth: an observational study protocol

**DOI:** 10.1186/s12884-023-05824-x

**Published:** 2023-08-03

**Authors:** Eleanor M Scott, Helen R. Murphy, Jenny Myers, Ponnusamy Saravanan, Lucilla Poston, Graham R Law

**Affiliations:** 1 Leeds Institute of Cardiovascular and Metabolic Medicine, School of Medicine, LIGHT Laboratories, University of Leeds, Clarendon Way, Leeds, LS2 9JT UK; 2 Norwich Medical School, University of East Anglia, Norwich, UK; 3 Maternal and Fetal Health Research Centre, University of Manchester, Manchester, UK; 4 Populations, Evidence and Technologies, Division of Health Sciences, Warwick Medical School, University of Warwick, Warwick, UK; 5 Diabetes, Endocrinology and Metabolism, George Eliot Hospital, Nuneaton, UK; 6 Tommy’s Maternal and Fetal Research Unit, Kings College London, London, UK; 7 University of Lincoln, Lincoln, UK

**Keywords:** Gestational diabetes, Continuous glucose monitoring, Glycemia, Pregnancy, Fetal growth, Large for gestational age, Early diagnosis

## Abstract

**Background:**

Continuous glucose monitoring (CGM) provides the most objective method of assessing glucose in daily life. Although there have been small, short-term physiologic studies of glucose metabolism in ‘healthy’ pregnant women a comprehensive, longitudinal description of changes in glucose over the course of pregnancy and how glucose dysregulation earlier in pregnancy relates to traditional third trimester screening for gestational diabetes, fetal growth and pregnancy outcomes is lacking. This study aims to characterise longitudinal changes in glycemia across gestation using CGM, in order to understand the evolution of dysglycemia and its relationship to fetal growth.

**Method/design:**

A multi-centre, prospective, observational, cohort study of 500 healthy pregnant women, recruited in the first trimester of pregnancy. Masked CGM will be performed for a 14-day period on five occasions across pregnancy at ~ 10–12, 18–20, 26–28, 34–36 weeks gestation and postnatally. Routinely collected anthropometric and sociodemographic information will be recorded at each visit including: weight, height, blood pressure, current medication. Age, parity, ethnicity, smoking will be recorded. Blood samples will be taken at each visit for HbA1c and a sample stored. Details on fetal growth from ultrasound scans and the OGTT results will be recorded. Maternal and neonatal outcomes will be collected. CGM glucose profiling is the exposure of interest, and will be performed using standard summary statistics, functional data analysis and glucotyping. The primary maternal outcome is clinical diagnosis of GDM. The primary neonatal outcome is large for gestational age (LGA) (> 90th centile defined by customised birthweight centile). The relationship of glucose to key secondary maternal and neonatal outcomes will be explored.

**Discussion:**

This study will ascertain the relationship of maternal dysglycemia to fetal growth and outcomes. It will explore whether CGM glucose profiling can detect GDM before the OGTT; or indeed whether CGM glucose profiling may be more useful than the OGTT at detecting LGA and other perinatal outcomes.

**Trial registration:**

ISRCTN 15,706,303 https://www.isrctn.com/ISRCTN15706303 Registration date: 13th March 2023.

## Background

The UK has one of the highest rates of stillbirth and neonatal death in Europe [1]. The MBRRACE-UK report identified that half of mothers with a perinatal death had an abnormality of fetal growth [1]. Furthermore, infants who are born large or small for gestational age (LGA or SGA respectively) are predisposed to developing obesity and Type 2 Diabetes (T2DM) perpetuating an intergenerational cycle of cardiometabolic disease [2–4].

Amongst the many factors that influence fetal growth, maternal glycemic control plays a key role and is a modifiable risk factor. Maternal hyperglycaemic excursions stimulate fetal insulin secretion leading to increased fetal adiposity and growth [5]. This increases the risk of preterm and instrumental delivery, neonatal hypoglycaemia requiring neonatal intensive care unit (NICU) admission, caesarean section, stillbirth. Difficulties at delivery can lead to brain damage, shoulder dystocia and permanent disability [6, 7]. Observational studies demonstrate that varying degrees of dysglycemia during pregnancy, including pre-existing diabetes and gestational diabetes (GDM) are associated with LGA related adverse perinatal outcomes [8, 9].

To mitigate against hyperglycemia related fetal growth and associated adverse pregnancy outcome, clinical guidelines currently recommend testing for pregnancy related dysglycemia at 24–28 weeks gestation by an oral glucose tolerance test (OGTT) [10]. NICE recommends diagnosis of GDM on the basis of a raised fasting or 2 h glucose. This late pregnancy diagnosis of GDM is an increasing cause for concern; we have shown that excess fetal growth assessed by ultrasound scan is detectable from 20 weeks’ gestation, pre-dating biochemical diagnosis of GDM [11, 12]. Whilst we now know that testing at 24–28 weeks gestation is too late to prevent abnormal fetal growth, there is no validated test for earlier diagnosis and the optimal time of screening and treatment for dysglycemia remains unknown [13]. Indeed, despite OGTT testing, the majority of LGA babies are born to mothers without a diagnosis of GDM [8, 14, 15]. This concurs with the recognition that the OGTT is insufficiently sensitive and that many women who could benefit from treatment will not be correctly identified, contributing to the failure to improve outcomes. The OGTT, recognised to be an outdated test for diabetes diagnosis, is now used only in pregnancy. It is known to be poorly reproducible: 40% of pregnant women who had a 2nd OGTT immediately after an abnormal OGTT had normal results and vice-versa [16].

Glucose control is dynamic, with glucose tolerance and insulin sensitivity varying across the 24 h day with a circadian rhythm [17, 18]. Insulin sensitivity also varies across pregnancy, with insulin resistance increasing with gestation [19]. As the OGTT relies on just two glucose readings taken two hours apart on one day, it cannot detect the nuances of daily glycemic variations, or changes across pregnancy.

Continuous glucose monitoring (CGM) provides the most objective method of assessing glucose in daily life [20]. With up to 288 interstitial fluid glucose measurements per day and > 4000 measures over a single 2-week sensor life-span, CGM accurately reflects blood glucose variations [20]. Although there have been small, short-term physiologic studies of glucose metabolism in ‘healthy’ pregnant women [19, 21–23] a comprehensive, longitudinal description of changes in glucose over the course of pregnancy and how glucose dysregulation earlier in pregnancy relates to traditional 3rd trimester GDM screening, fetal growth and pregnancy outcomes is lacking. The availability of non-burdensome, CGM technology and intensive glucose profiling techniques provides a new opportunity to accomplish this goal.

Building on our earlier work [24–31] we hypothesise that intensive glucose profiling of CGM will be able to detect maternal glucose dysregulation early in pregnancy that current clinical testing does not detect and that this will be associated closely with the development of LGA and adverse pregnancy outcomes.

## Methods / design

### Primary research questions

1. To characterise longitudinal changes in glycemia across gestation and into the postnatal period, by obtaining detailed CGM glucose summary statistics, functional data analysis profiles and glucotype at multiple time points.
2. To determine the relationship between CGM glucose measures, fetal growth and pregnancy outcomes.
3. To determine the relationship of the CGM glucose measures to the OGTT and HbA1c.

#### Study design

A multi-centre, prospective observational cohort study.

#### Population

500 healthy pregnant women in the first trimester of pregnancy.

#### Eligibility criteria

Women aged 18–45 years with a singleton pregnancy who are classified as at risk for GDM with at least 1 of the following risk factors: BMI > 30 kg/m2; previous unexplained still birth or baby > 4.5 kg; first degree relative with diabetes; ethnic minority group (e.g. South Asian, Middle-Eastern, Afro-Caribbean).

#### Exclusion criteria

Women presenting beyond 14 weeks gestation; T1DM or T2DM; previous GDM; women on metformin therapy for infertility; multiple pregnancy, fetal congenital abnormality; significant co-existent medical or psychiatric conditions; unable to understand English and no translator available.

#### Recruitment

Participants will be recruited from three major Hyperglycemia in Pregnancy NHS services across the UK. Pregnant women will be invited to participate in the study at their routine clinical dating scan (10–12 weeks), having been sent written information about the study prior to this.

#### Study visits and data collection

Those who consent to take part will wear CGM for a fortnight on five occasions at ~ 10–12, 18–20, 26–28, 34–36 weeks gestation and immediately postnatally (to coincide with routine clinic attendance to minimise burden and to maximise participation/concordance where possible), see Table 1.

**Table 1:** Spirit table

Summary of information collected		Visit 1	Visit 2	Visit 3	Visit 4	Visit 5
**Gestation (weeks)**		10–12	18–20	26–28	34–36	Immediate postnatal
**Enrollment**	Eigibility screen	x				
	**Consent**	x				
**Maternal**	Demographics	x				
	BMI	x	x	x	x	x
	BP	x	x	x	x	x
	Pregnancy outcome and complications					x
**Laboratory**	OGTT			x		
	HbA1c	x	x	x	x	x
	Blood stored	x	x	x	x	x
	Rectal swab			x		
	Placenta and cord blood stored					x
**CGM (2 weeks)**		x	x	x	x	x
**Neonatal**	Birthweight					x
	Anthropometry					x

Routinely collected anthropometric and sociodemographic information will be recorded at each visit including: weight, height, blood pressure, current medication. Age, parity, ethnicity, smoking will be recorded at the first visit. Blood samples will be taken at each visit for HbA1c and a sample stored (for subsequent analysis to identify mechanisms underlying dysglycemia, as well as predictors of GDM and fetal growth). The research midwife will insert the CGM sensor and the participant will return it via stamped addressed envelope for data download. Details on fetal growth from ultrasound scans (USS) and the OGTT results will be recorded. A rectal swab will be requested (optional) at 26–28 weeks gestation and stored for microbiome analysis.

Post-delivery, placenta and cord blood samples will be collected and stored in designated Human Tissue Act approved and compliant facilities for later analysis. Infant anthropometry measures will be taken (skin fold thickness). Maternal and neonatal outcomes will be collected as detailed below.

##### CGM measurement

A small factory calibrated, masked (patient cannot see the data) CGM device, (Abbott Diabetes Care) will be used. It is a small discreet, water resistant sensor, with a very thin filament (< 0.4 mm thick) that is inserted (painlessly) 5 mm beneath the skin surface on the upper arm. It is factory calibrated, making it far more convenient and removing the risk for sensor inaccuracies from calibration user error (capillary meter inaccuracy, not washing hands etc.) seen with other CGM devices. A single reader device (at each centre) is used to activate and retrieve the data. The accuracy and safety of this device has been established in pregnant women across all trimesters, including those with HbA1c levels indicating euglycaemia [32] The accuracy has been established across the full range of glucose levels likely to be observed in this study, including those in the normal glucose range. Importantly, it is extremely acceptable to pregnant women and is CE marked and fully approved for use in pregnancy [32].

##### CGM glucose profiling

Standard summary statistics of the CGM data for each of the five measurement periods will be calculated, including: mean daily/weekly CGM glucose; percentage of time spent within, above, and below the glucose target range of 3.5–7.8 mmol/l; area under the curve (a measure of participants’ exposure to high, low and normal glucose levels over time) for all glucose measurements that exceed thresholds of 7.8 mmol/l or 6.7 mmol/l, and fall below thresholds of 3.5 mmol/l or 2.8 mmol/l; number of high/low glucose excursions; glycaemic variability [20]. Functional data analysis will be performed as previously detailed to generate temporal profiles for each measurement period [24, 29–31]. Glucotyping will be performed as previously described [33].

##### Other glucose measures

The blood glucose values obtained in response to 75 g OGTT at 24–28 weeks’ gestation will be recorded at 0 and 120 min. HbA1c will be analysed to DCCT standards.

##### Assessment of fetal growth

Infant birthweight will be recorded. LGA will be defined as birthweight > 90th centile and SGA < 10th centile, using customised GROW birthweight centiles, (GROW@perinatal.org) which adjusts for maternal height, weight, ethnicity, parity, neonatal sex and gestational age. Fetal anthopometry measures, including sum of skin folds and neonatal fat mass, will be taken after delivery. USS are performed routinely in all pregnant women at 12 weeks (dating scan) and 20 weeks (anatomy scan) gestation and in women diagnosed with GDM growth scans are performed at 28, 32 and 36 weeks gestation [10]. Fetal biometry and estimated fetal weight will be obtained from each.

##### Maternal and neonatal pregnancy outcomes

The following outcomes will be recorded: Maternal - hypertensive disorders (chronic; gestational; pre-eclampsia); mode of delivery inc. caesarean section; maternal weight gain; maternal length of stay; Neonatal - pregnancy loss < 20 weeks; stillbirth; termination; congenital anomaly; preterm births (< 37 weeks; <34 weeks) gestational age at delivery; neonatal hypoglycaemia, neonatal ICU admission, birth injury, shoulder dystocia, hyperbilirubinemia, respiratory distress; infant length of stay; composite neonatal outcome.

### Exposure

CGM glucose is the exposure of interest (summary statistical measures, mean FDA glucose and glucotype).

### Primary outcome

The primary maternal outcome is a clinical diagnosis of GDM, and the primary neonatal outcome is Large for Gestational Age (LGA) at birth (> 90th centile defined by customised birthweight centile).

### Secondary outcomes

Secondary outcomes include other measures of fetal growth (to include: birthweight; SGA on GROW centile; LGA and SGA defined on Intergrowth and WHO centiles; neonatal adiposity at birth; USS measures of abdominal circumference, estimated fetal weight at 28, 32 and 36 weeks gestation) and the range of maternal and neonatal outcomes as above.

### Statistical analysis plan

A Directed Acyclic Graph will be developed to direct the modelling of the data to correctly identify confounders, mediators and competing exposures [34].

Detailed CGM glucose summary statistics, functional data analysis profiles and glucotype will be generated for both weeks of each of the five measurement periods (a minimum of 96 h CGM data at each visit will be needed to be included in the analysis). Means ± SD values or percentiles appropriate to the variable under consideration will be reported for the covariates in relation to the outcome. Primary analysis will initially retain the measures of glucose (as the primary exposure of interest) distinct at different time of gestation. The modelling will adjust for week of gestation, and is already adjusted for maternal height, weight, ethnicity, parity, neonatal sex using the GROW centile. Selected CGM outcomes will also be calculated to compare the between group differences. For assessing group differences in secondary outcomes of fetal growth measures and maternal and neonatal outcomes, regression models will be used to compare continuous and ordinal variables, logistic regression will be used to compare categorical variables, and Poisson regression models will be used to compare event rates. Missing data for outcomes will not be imputed. Available case analysis method will be used for secondary outcome analyses in each time point.

Functional Data Analysis: The life course nature of the data means that, for any individual, the measures will be more correlated than those measures between individuals. A functional multilevel model will be used to reflect the complex structure of the data, as well as covariance and correlations therein, to correctly infer statistical relevance within the model. Point estimates, 95% confidence intervals and p-values will be reported for the average effect at each time point of CGM measurement. Residual values will be examined for an approximate normal distribution. If values are highly skewed then a transformation or robust statistical methods will be used instead.

#### Sample size estimation

The sample size calculations are based on two of the key findings from our pilot data: (1) mean CGM glucose; (2) glucotype. For the first a sample size of 280 women, will give 28 women who have LGA and 252 who do not, with a 90% power to detect a difference in glucose of 0.4 mmol/l with a SD of 0.6 at p = 0.05. This is a clinically relevant effect size in pregnancy. This has been illustrated in HAPO [8] with an OR 1.38 (1.32–1.44) for birthweight > 90th centile, with an increase in glucose of 0.4 mmol/l; A similar effect size was seen in CONCEPTT [26] and previous CGM analysis in pre-gestational diabetes [24, 29, 30]. Furthermore, improving CGM glucose by this small, but clinically relevant, amount in pregnancy is significantly associated with improved clinical outcomes, including LGA [26]. To ascertain the exposure of severe glucotype to LGA a sample size of 384 women, will give 38 women with LGA, and 346 who do not, with 90% power to detect a difference in glucotype (based on the prevalence of severe glucotype in the non LGA group of 37%, and in the LGA group of 64%). As GDM diagnosed by OGTT has a prevalence of 10%, then the sample sizes for this outcome, with these two exposures, are the same as for LGA. Allowing for ~ 30% drop out (including miscarriage and/or incomplete data capture) 500 recruits will be needed.

#### Trial management

University of Leeds is the study sponsor and has delegated responsibility for the overall management of the study to the chief investigator, including the design, coordination, monitoring and analysis and reporting of results. A Study Steering Committee SSC has been set up to assist with developing the design, co-ordination and strategic management of the study.

#### Data management

Data and statistical analyses will be handled by the University of Lincoln, in conjunction with the University of Leeds, United Kingdom.

## Discussion

This study will provide an extensive number of individual glucose measurements which will ascertain the relationship of maternal dysglycemia to fetal growth and outcomes. It will explore whether CGM glucose profiling can detect GDM before the OGTT; or indeed whether CGM glucose profiling may be more useful than the OGTT at detecting LGA and other perinatal outcomes. This data will be relevant to inform future NICE and International guidelines on how best to screen and detect GDM using CGM.

## Data Availability

Requests for access to study data and stored samples will be considered, and approved in writing where appropriate, after formal application to the SSC. Inquiries for data should be addressed to the principal investigator Prof Eleanor M Scott.

## List of abbreviations

BMI: Body Mass Index
CGM: Continuous glucose monitoring
FDA: Functional data analysis
GDM: Gestational diabetes
GROW: Gestation related optimal weight
HbA1c: Haemoglobin A1c
HAPO: Hyperglycaemia and adverse pregnancy outcomes
HRA: Health research authority
LGA: Large for gestational age
NICE: National institute for heath and care excellence
NHS: National health service
NICU: Neonatal intensive care unit
OR: Odds ratio
OGTT: Oral glucose tolerance test
SD: Standard deviation
SGA: Small for gestational age
SSC: Study steering committee
T2DM: Type 2 diabetes mellitus
T1DM: Type 1 diabetes mellitus
USS: Ultrasound scan
